# Evaluation of the Cepheid Xpert *C. difficile* diagnostic assay: an update meta-analysis

**DOI:** 10.1007/s42770-021-00563-7

**Published:** 2021-08-29

**Authors:** Yuanyuan Bai, Yingying Hao, Zhen Song, Wenjun Chu, Yan Jin, Yueling Wang

**Affiliations:** Department of Clinical Laboratory, Shandong Provincial Hospital Affiliated to Shandong First Medical University, 324 Jingwu Road, Jinan, People’s Republic of China

**Keywords:** Nucleic acid amplification techniques, *Clostridium* infections, Meta-analysis

## Abstract

**Background:**

Accurate and rapid diagnosis of *Clostridium difficile* infection (CDI) is critical for effective patient management and implementation of infection control measures to prevent transmission.

**Objectives:**

We updated our previous meta-analysis to provide a more reliable evidence base for the clinical diagnosis of Xpert *C. difficile* (Xpert *C. difficile*) assay.

**Methods:**

We searched PubMed, EMBASE, Cochrane Library, Chinese National Knowledge Infrastructure (CNKI), and the Chinese Biomedical Literature Database (CBM) databases to identify studies according to predetermined criteria. STATA 13.0 software was used to analyze the tests for sensitivity, specificity, positive likelihood ratio, negative likelihood ratio, diagnostic odds ratio, and area under the summary receiver operating characteristic curves (AUC). QUADAS-2 was used to assess the quality of included studies with RevMan 5.2. Heterogeneity in accuracy measures was tested with Spearman correlation coefficient and *chi*-square. Meta-regressions and subgroup analyses were performed to figure out the potential sources of heterogeneity. Model diagnostics were used to evaluate the veracity of the data.

**Results:**

A total of 26 studies were included in the meta-analysis. The pooled sensitivity (95% confidence intervals [CI]) for diagnosis was 0.97(0.95–0.98), and specificity was 0.96(0.95–0.97). The AUC was 0.99 (0.98–1.00). Model diagnostics confirmed the robustness of our meta-analysis’s results. Significant heterogeneity was still observed when we pooled most of the accuracy measures of selected studies. Meta-regression and subgroup analyses showed that the sample size and type, ethnicity, and disease prevalence might be the conspicuous sources of heterogeneity.

**Conclusions:**

The up-to-date meta-analysis showed the Xpert CD assay had good accuracy for detecting CDI. However, the diagnosis of CDI must combine clinical presentation with diagnostic testing to better answer the question of whether the patient actually has CDI in the future, and inclusion of preanalytical parameters and clinical outcomes in study design would provide a more objective evidence base.

## Introduction

*Clostridioides (Clostridium) difficile* infection (CDI) is the leading cause of healthcare-associated infections in the USA and is responsible for approximately 15,000 deaths annually in the USA [1, 2]. It accounts for 15 to 25% of healthcare-associated diarrhea cases in all healthcare settings [3]. Acquisition of *C. difficile* as a healthcare-associated infection (HAI) is associated with increased morbidity and mortality. It has been estimated that the length of CDI-associated hospital stays has increased and the average cost per case for HAI is more than $30,000 which is 1.5 times the cost of community-associated CDI (CAI) [4].

The clinical signs and symptoms presented by CDI are highly nonspecific, making it difficult to differentiate CDI from non-CDI, including non-CDI diarrhea in a *C. difficile*-colonized patient [5]. Therefore, accurate diagnosis of CDI is critical for effective patient management and implementation of infection control measures to prevent transmission [6]. Diagnostic tests for *C. difficile* are classified as tests for *C. difficile* products (GDH, TcdA, and TcdB), toxigenic culture methods (TC), cell cytotoxicity neutralization assay (CCNA), toxin detection by enzyme immunoassays (EIA), and detection of toxin genes by nucleic acid amplification tests (NAATs). However, the best practices for laboratory diagnosis of CDI remain controversial [7]. The anaerobic toxigenic culture (TC) and culture cytotoxicity neutralization assay (CCNA) were often used as the laboratory reference tests for detecting *C. difficile*. Unfortunately, both tests are slow and labor-intensive [8]. EIAs for toxins A and B are rapid and relatively inexpensive, but it was ultimately demonstrated that EIAs cannot be used as stand-alone tests due to their low sensitivity [9]. Although the accurate and rapid diagnosis of CDI is essential for effective and timely treatment, this remains an unmet clinical need.

Currently, several NAATs have been cleared by the Food and Drug Administration (FDA) [10] and supported by recent guidelines by the American Society of Microbiology [11]. We previously published a meta-analysis to evaluate the Xpert *C. difficile* system (Cepheid, USA), a platform that detects the toxin B gene (*tcd*B), the CDT component A gene (*cdt*A), and a deletion within the LCT regulatory gene *tcd*C to putatively identify “hypervirulent” RT 027 [12]. These strains have been shown to produce a large amount of toxins in vitro and are associated with erythromycin and newer fluoroquinolones resistance. The Xpert *C. difficile* assay is among the simplest to perform and, with a turnaround time of about 1 h, is also the most rapid of the NAATs available. Since the publication of the previous meta-analysis, four new researches including 1141 patients that evaluated the diagnostic accuracy of Xpert *C. difficile* assay have been published.

As the latest 2018 guidelines from the IDSA recommend that NAATs can be used as a stand-alone diagnostic test in cases where there are pre-agreed institutional criteria for patient stool submission [13], we identified the need to update our 2017 meta-analysis to provide a more reliable evidence base for the clinical application of Xpert *C. difficile* assay, and the results are presented in this report.

## Materials and methods

We followed the Preferred Reporting Items for Systematic Reviews and Meta-Analyses (PRISMA) guidelines in our study.

## Literature search

PubMed, EMBASE, Cochrane Library databases, Chinese National Knowledge Infrastructure (CNKI), and Chinese Biomedical Literature Database (CBM) were searched from July 2016 to the end of April 2020 without language restrictions by two investigators (YY Bai and YL Wang). The search terms used were as follows: *Clostridium difficile* AND (Xpert *C. difficile* OR molecular diagnostic techniques). Reference lists from included studies were also searched. We reviewed and included our previous search and added all relevant articles, focusing on the time after the previous search.

## Study criteria

We searched the literature using the following predetermined inclusion criteria. Studies evaluating Xpert CD as a diagnostic test for CDI were eligible for inclusion if the studies (1) described original research; (2) performed stool samples analyses from human patients, either children or adults; (3) compared Xpert CD to a reference method — either CCNA or anaerobic TC; and (4) had extractable data to fill the 4 cells of a 2 × 2 table for diagnostic tests (true positives (TP), true negatives (TN), false positives (FP), and false negatives (FN)).

Relevant publications were excluded if they were duplicated articles, letters without original data, animal studies, case reports, editorials, and reviews. Studies with fewer than 20 samples were also excluded to reduce selection bias. Articles that contain data from infants were excluded because infants rarely develop clinical infection.

## Data extraction

Two investigators (YY Bai and YL Wang) extracted data from full text of the included studies independently. Disagreements were resolved by consensus. Information was extracted on the first author, publication year, country where the study was conducted, sample size, reference tests the diagnosis used, the number of TP, the number of FP, the number of FN, and the number of TN. These were summarized as sensitivity, TP/(TP + FN); specificity, TN/(TN + FP); and prevalence, (TP + FN)/(TP + FN + TN + FN).

## Quality of study reports

We applied the Quality Assessment of Diagnostic Accuracy Studies (QUADAS-2) to assess the quality of included studies (http://www.bris.ac.uk/quadas/), an updated version of the original software [14].

## Statistical analysis

### Accuracy estimates

Meta-analyses were performed using two software programs: STATA 13.0 (Stata Corporation, Texas, USA) and Cochrane RevMan 5.2. Sensitivity, specificity, positive likelihood ratio (PLR), negative likelihood ratio (NLR), diagnostic odds ratio (DOR), forest plots, and summary receiver operating characteristic (SROC) curves were analyzed with the “MiDAS” module for STATA 13.0, based on the random model effect. Quality of studies was assessed with RevMan 5.2.

### Heterogeneity

We used chi-square test and *I*^2^ (*p* < 0.05 and *I*^2^ > 50% indicated significant heterogeneity) to identify heterogeneity. Heterogeneity was evaluated using the methods detailed in our previous study [15]. To assess the potential sources of heterogeneity, we further performed subgroup analyses and meta-regressions according to the descriptions of the included studies. Model diagnostics were used to evaluate the veracity of the data. Extreme outliers and highly influential cases were reevaluated and corrected as described above if appropriate. Deeks’ funnel plot asymmetry test was performed to investigate the publication bias, with *p* < 0.10 showing significant publication bias, which is an important concern for meta-analyses of diagnostic accuracy [16].

## Results

### Characteristics of the selected studies

**Fig. 1:**
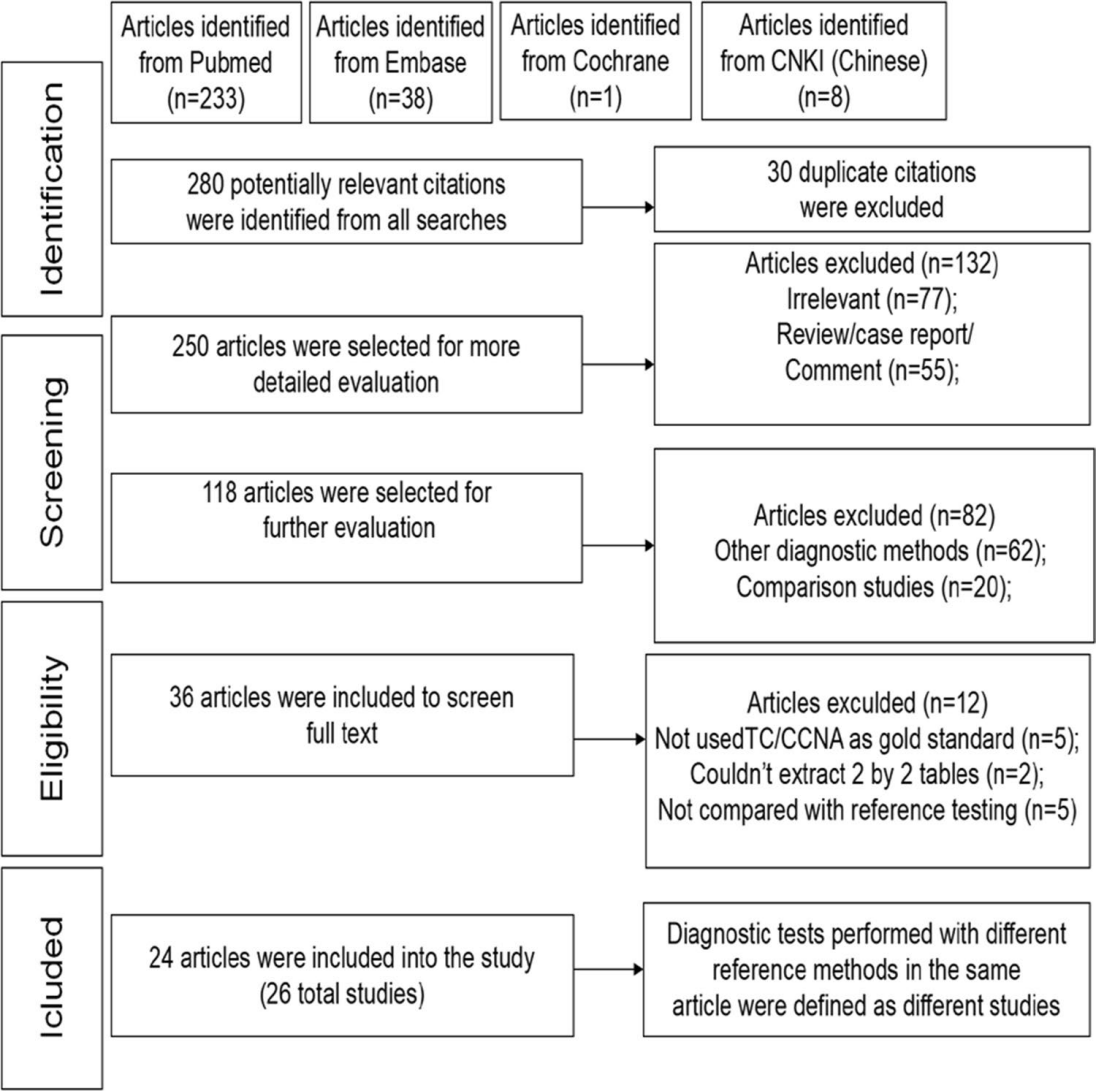
Flow chart of study selection

A flow chart of the study selection process is shown in Fig. 1. A total of 280 potentially relevant citations were identified from all searches. Finally, according to the inclusion and exclusion criteria, since the publication of our previous meta-analysis, four new Asian-based studies involving 1141 additional patients were included in this updated meta-analysis. Because diagnostic tests performed with different reference methods occurred in the same article, 26 independent studies (including 10,493 samples) were defined in the meta-analysis. Table 1 shows the characteristics of these included studies [17–40]. Most of the studies were prospective in design, but only 10 studies were blind (data not shown).

**Table 1:** Summary of the included studies

First author	Year	Country	Sample size	Reference test	Specimen type	TP ^1)^	FP^2)^	FN^3)^	TN^4)^	Calculated prevalence (%)
Huang [17]	2009	USA	220	CCNA^5)^	Fresh stools	34	13	1	172	15.9
Tenover-1 [18]	2010	Canada and USA	2296	TC^6)^	Fresh stools	245	188	3	1860	10.8
Tenover-2 [18]	2010	Canada and USA	2296	Enriched TC	Fresh stools	316	117	22	1841	14.7
Novak-Weekley [19]	2010	USA	428	Enriched TC	Fresh stools	68	13	4	343	16.8
Swindells-1 [20]	2010	UK	150	CCNA	Fresh stools	15	4	0	131	10
Swindells-2 [20]	2010	UK	150	TC	Fresh stools	19	1	0	130	12.7
Goldenberg [21]	2010	UK	224	TC	Fresh stools	57	6	0	161	25.4
Dubberke [22]	2011	USA	150	TC	Frozen stools	44	7	0	99	29.3
Zidaric [23]	2011	Slovenia	178	TC	Frozen stools	27	4	1	146	15.7
Buchan [24]	2012	USA	275	TC	Fresh stools	58	18	0	199	21.1
Viala [25]	2012	France	94	TC	Fresh stools	44	1	1	48	47.9
Shin [26]	2012	Korea	248	TC	Fresh stools	49	10	0	189	19.6
Dalpke [27]	2013	Germany	448	TC	Fresh stools	72	8	2	366	16.5
Eigner [28]	2014	Germany	245	TC	Fresh stools	74	8	2	161	31
Gilbreath [29]	2014	USA	190	TC	Frozen stools	23	2	0	165	12.1
Jensen [30]	2015	Denmark	299	TC	Fresh stools	38	20	0	241	16.6
Jazmati [31]	2015	Germany	199	Enriched TC	Frozen/fresh stools	28	17	0	154	14.1
Yoo [32]	2015	Korea	254	TC	Frozen/fresh stools	72	2	15	165	34.1
Moon [33]	2016	Korea	258	TC	Frozen/fresh stools	52	11	3	192	21.3
Moon [34]	2016	Korea	270	TC	Fresh stools	52	11	3	204	20.4
Shin [35]	2016	Korea	339	TC	Frozen/fresh stools	78	18	9	234	25.7
Rajabally [36]	2016	South Africa	141	TC	Fresh stools	27	3	3	108	21.3
Paitan [37]	2017	Israel	209	TC	Frozen stools	97	0	1	111	46.9
Seo [38]	2017	Korea	190	TC	Fresh stools	33	0	2	155	18.4
Huang [39]	2017	China	43	TC	Frozen/fresh stools	10	2	1	30	25.6
Wu [40]	2019	China	699	TC	Fresh stools	144	37	9	509	22.0

^1^*TP*, true positives; ^2^*FP*, false positives; ^3^*FN*,false negatives; ^4^*TN*, true negatives; ^5^*CCNA*, culture cytotoxicity neutralization assay; ^6^*TC*, toxigenic culture

### Quality assessment

**Fig. 2:**
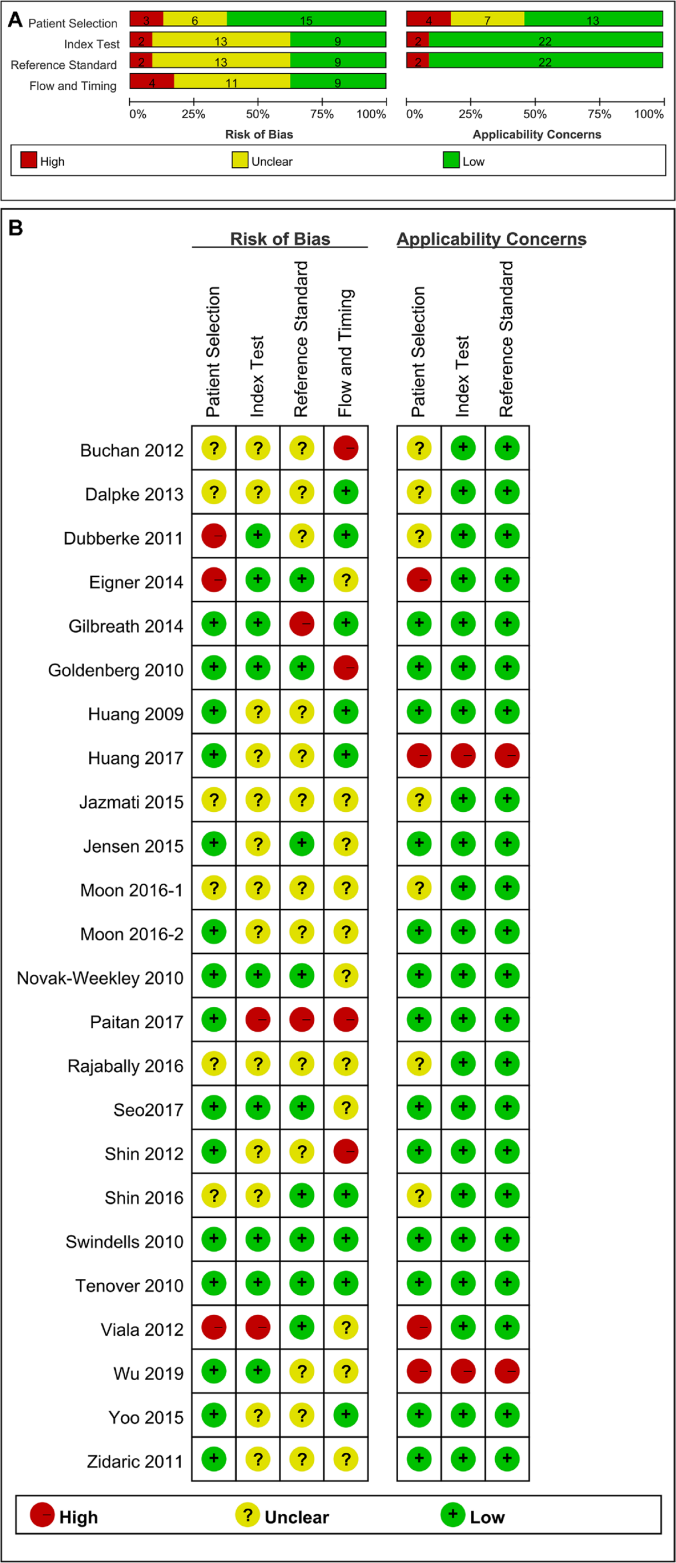
Quality assessment of included studies

A quality assessment of all the included articles is illustrated in Fig. 2. In conclusion, patient selection provided the most high-risk bias and high-risk applicability concerns. Nearly half of the included articles were at either high-risk or unclear-risk bias in “patient selection” and “flow and timing” domains of QUADAS-2 due to the lack of detail regarding timing, inconsecutive, or nonrandom patient selection and blinding. A total of 13 (54%) studies were at low risk, 7 studies (29%) were of unclear risk, and 4 studies (17%) were at high risk for patient selection bias. Most of the articles provided either low or unclear risk in the index test and reference standard bias domains. Regarding applicability, half of the articles were at high risk for patient selection; however, most of the articles (*n* = 22, 91.7%) were in the low risk of index test and reference standard domains.

### Diagnostic accuracy

**Fig. 3:**
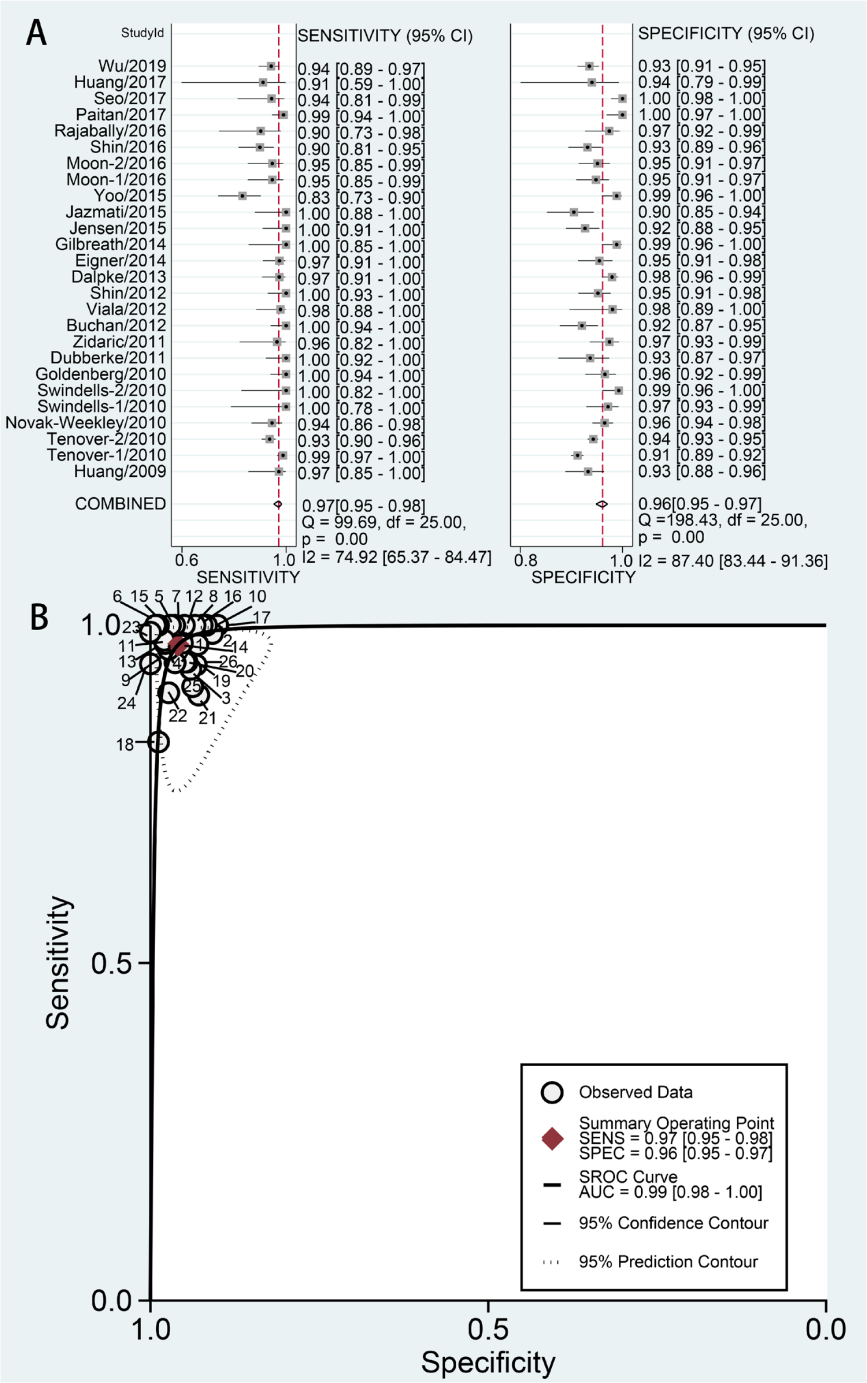
Forest plots of the pooled sensitivity and specificity and SROC curve of Xpert CD for detection of CDI. **a** Forest plots of the pooled sensitivity and specificity. Each solid square represents an individual study. Error bars represent 95% CI. Diamond indicates the pooled sensitivity and specificity for all of the studies. **b** SROC curve

Results are given as values (95% CI). Using a random-effects model, the results were as follows: sensitivity 0.97(0.95–0.98), *I*^2^ = 74.9%; specificity 0.96(0.95–0.97), *I*^2^ = 87.4% (Fig. 3); PLR 23.81(17.93–31.61), *I*^2^ = 82.27%; NLR 0.03 (0.02–0.05), *I*^2^ = 71.40%; DOR 784.85 (440.25–1399.16), *I*^2^ = 100%; and AUC 0.99 (0.98–1.00) (Fig. 3).

**Fig. 4:**
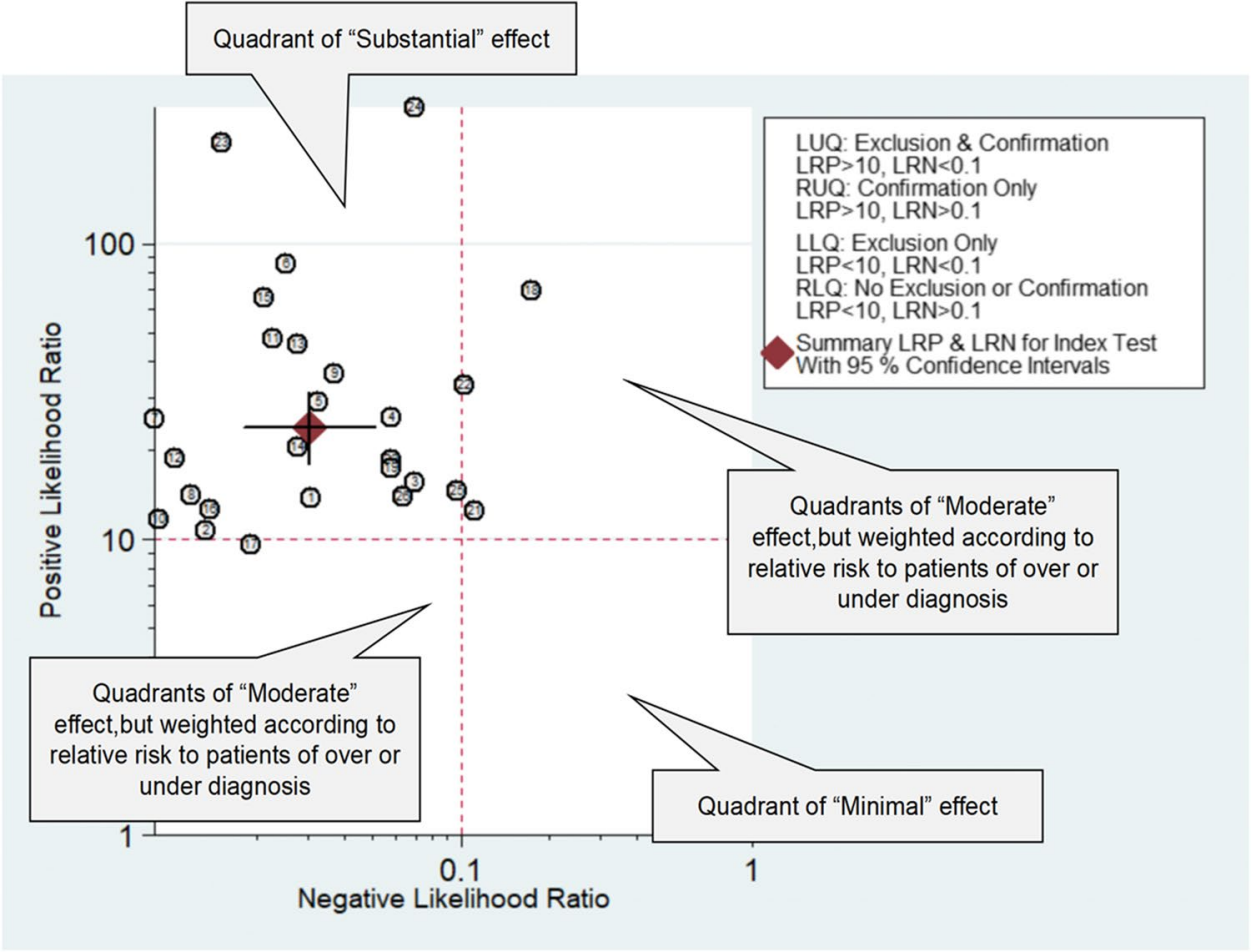
Using of the likelihood ratio scatter matrix to aid in the decision of effect size. *LUQ*, left upper quadrant; *RUQ*, right upper quadrant; *LLQ*, left lower quadrant; *RLQ*, right lower quadrant; *LRP*, positive likelihood ratio; *LRN*, negative likelihood ratio

To create an overall index of effect, the likelihood ratio scatter matrix was utilized. The paired likelihood ratios are within the areas that are typically used to indicate high clinical validity (+ LR of > 10 and -LR of < 0.1), the expert panel described this as a “substantial” effect, and the error bands of the estimate (as represented by the crosshairs on the summary diamond) do not cross into other quadrants (Fig. 4).

### Robustness tests

**Fig. 5:**
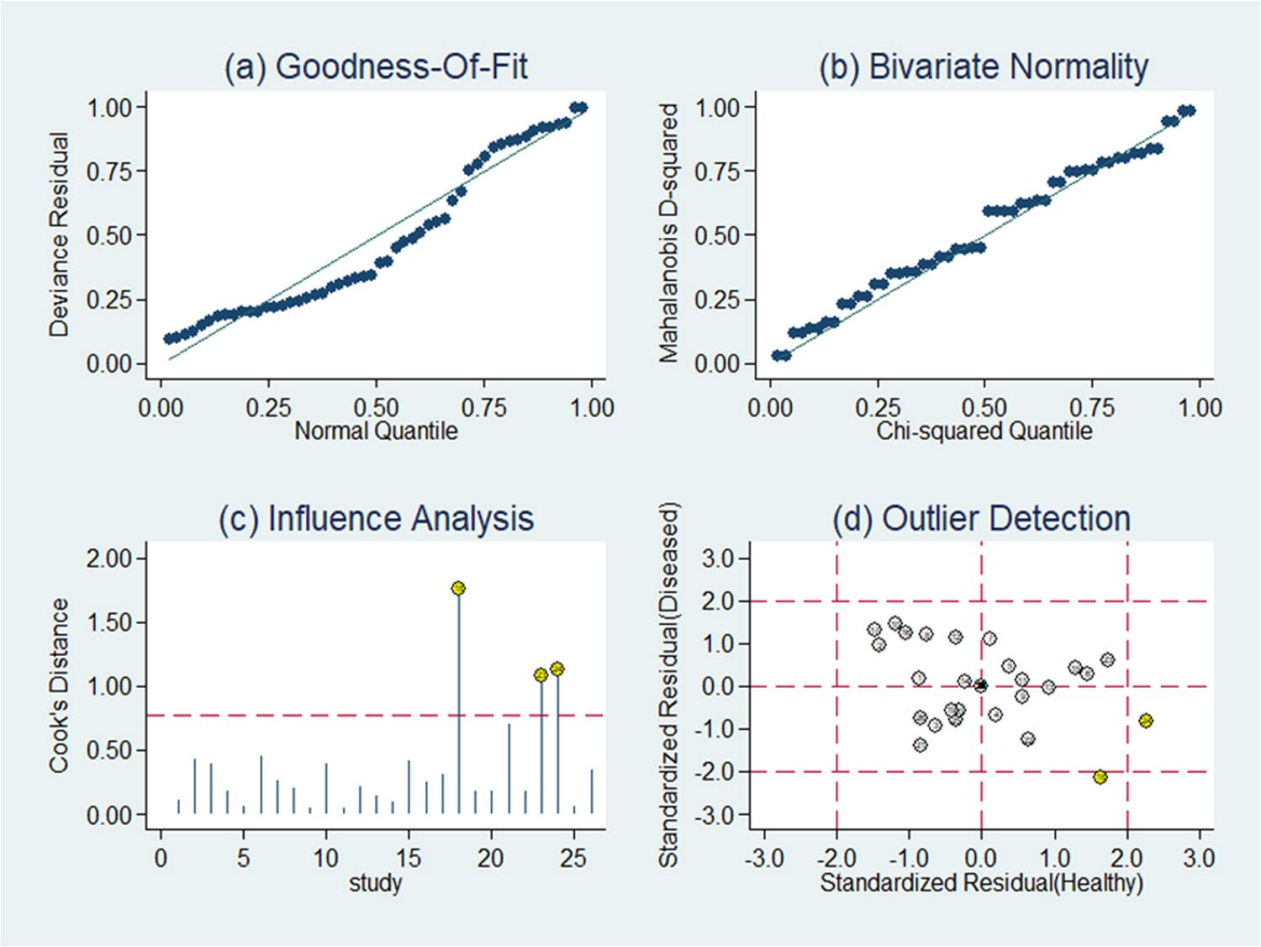
Graphs for sensitivity analyses: **a** goodness of fit, **b** bivariate normality, **c** influence analysis, and evaluation of Xpert detection system of Clostridium difficile outlier detection

**Fig. 6:**
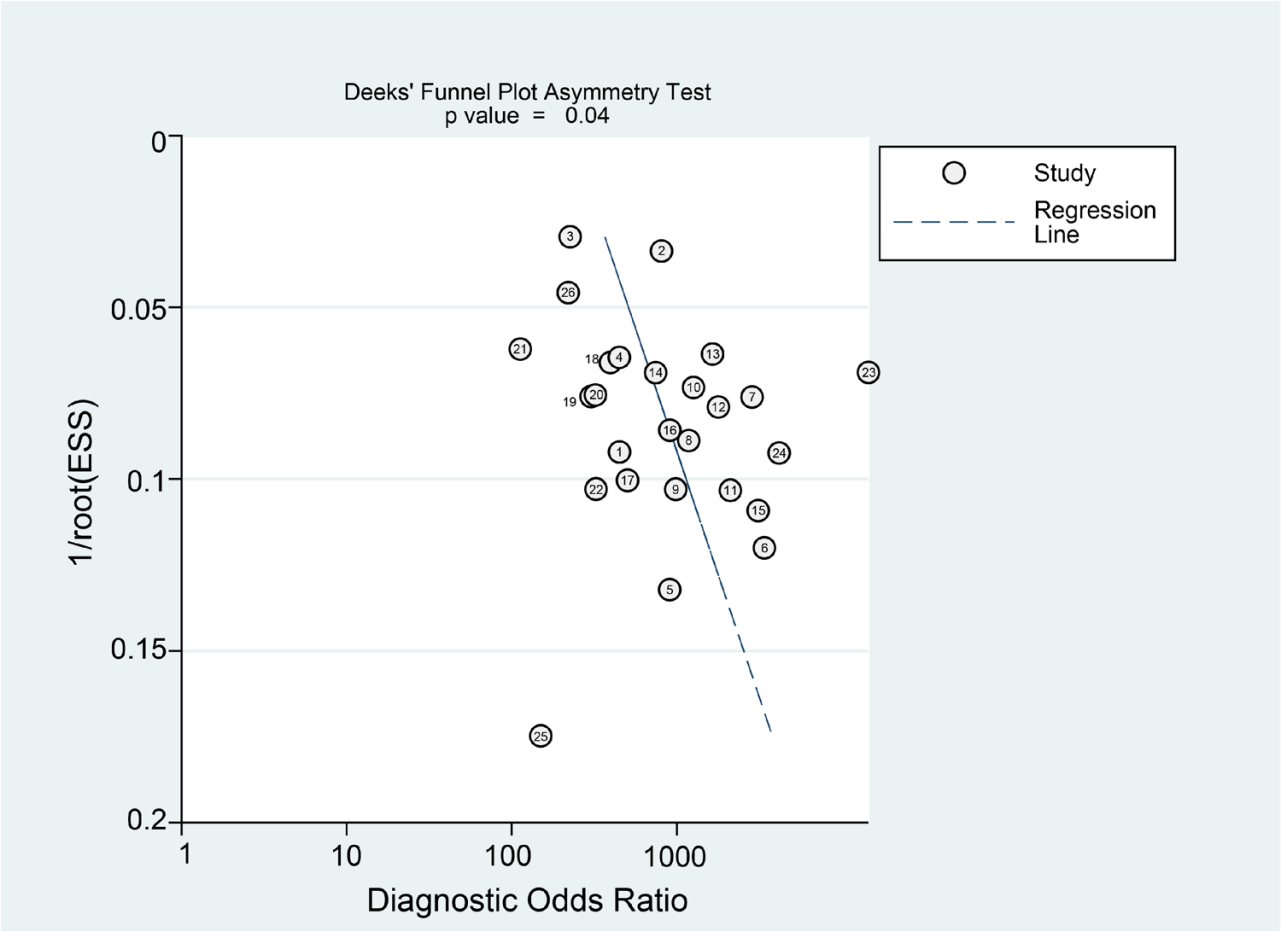
Graph of Deeks’ funnel plot asymmetry test

Goodness of fit and bivariate normality analyses (Fig. 5a, b) showed the bivariate model was moderately robust. Influence analysis and outlier detection identified only two outliers [18, 24] (Fig. 5c, d). Instructively, conducting the same analyses after excluding the two outliers did not significantly change the overall results (Table 2). Finally, the Deeks’ funnel plot asymmetry test was conducted to assess publication bias in this study (Fig. 6), which suggested statistically significant publication bias (*p* = 0.04).

**Table 2:** Subgroup analyses and Sensitive analyses

Analysis		No. of studies	Se (95% CI)	Sp (95% CI)	PLR (95% CI)	NLR (95% CI)	DOR (95% CI)	AUC (95%CI)
Overall		26	0.97(0.95–0.98)	0.96(0.95–0.97)	23.81(17.93–31.61)	0.03(0.02–0.05)	784.85(440.25–1399.16)	0.99 (0.98–1.00)
Outlier excluded		24	0.97(0.96–0.98)	0.95(0.94–0.96)	21.09(16.47–27.00)	0.03(0.02–0.05)	775.46(426.31–1410.60)	0.99 (0.98–1.00)
Subgroup								
Prevalence	< 15%	6	0.99(0.89–1.00)	0.96(0.92–0.98)	25.07(11.68–53.79)	0.01(0.00–0.13)	2672.14(141.68–50,398.07)	1.00(0.98–1.00)
	> 15%	20	0.97(0.94–0.99)	0.95(0.94–0.96)	20.20(16.20–25.19)	0.04(0.02–0.06)	553.43(306.69–998.69)	0.98(0.97–0.99)
Sample size	< 248	12	0.98(0.95–0.99)	0.96(0.94–0.98)	26.31(17.56–39.40)	0.02(0.01–0.05)	1212.88(477.04–3083.73)	1.00(0.98–1.00)
	> 248	14	0.96(0.93–0.98)	0.95(0.93–0.96)	17.55(13.72–22.47)	0.04(0.02–0.08)	400.20(228.74–700.18)	0.98(0.97–0.99)
Sample type	Fresh stools	17	0.97(0.95–0.98)	0.96(0.94–0.97)	22.15(16.34–30.02)	0.03(0.02–0.05)	733.13(402.34–1335.89)	0.99 (0.98–1.00)
	Frozen stools	9	0.97(0.91–0.99)	0.97(0.94–0.98)	27.73(14.91–51.55)	0.03(0.01–0.10)	809.74(231.64–2830.53)	0.99 (0.98–1.00)
Ethnicity	Asian	9	0.95(0.90–0.97)	0.97(0.94–0.98)	29.87(14.73–60.59)	0.06(0.03–0.10)	532.01(193.43–1463.21)	0.99 (0.98–1.00)
	Caucasian	17	0.98(0.96–0.99)	0.96(0.94–0.97)	21.89(16.10–29.76)	0.02(0.01–0.04)	1026.27(490.68–2146.47)	0.99 (0.98–1.00)

Abbreviations: *Se*, sensitivity; *Sp*, specificity; *PLR*, positive likelihood ratio; *NLR*, negative likelihood ratio; *DOR*, diagnostic odds ratio; *CI*, confidence interval; *AUC*, area under the curve

### Heterogeneity

There was substantial heterogeneity for all the statistical measures. The heterogeneity test results of sensitivity and specificity are illustrated in the forest plots (Fig. 3). The Spearman correlation coefficient between the logit of sensitivity and logit of 1-specificity was used to assess the threshold/cutoff effect. The Spearman correlation coefficient (*p* value) in diagnosis of CDI was 0.237 (*p* = 0.244). This indicated that the heterogeneity might not be due to threshold/cutoff effect. To assess for causes of variations other than threshold, we further performed meta-regressions and subgroup analyses according to the descriptions of the included studies.

### Meta-regression and subgroup analyses

**Fig. 7:**
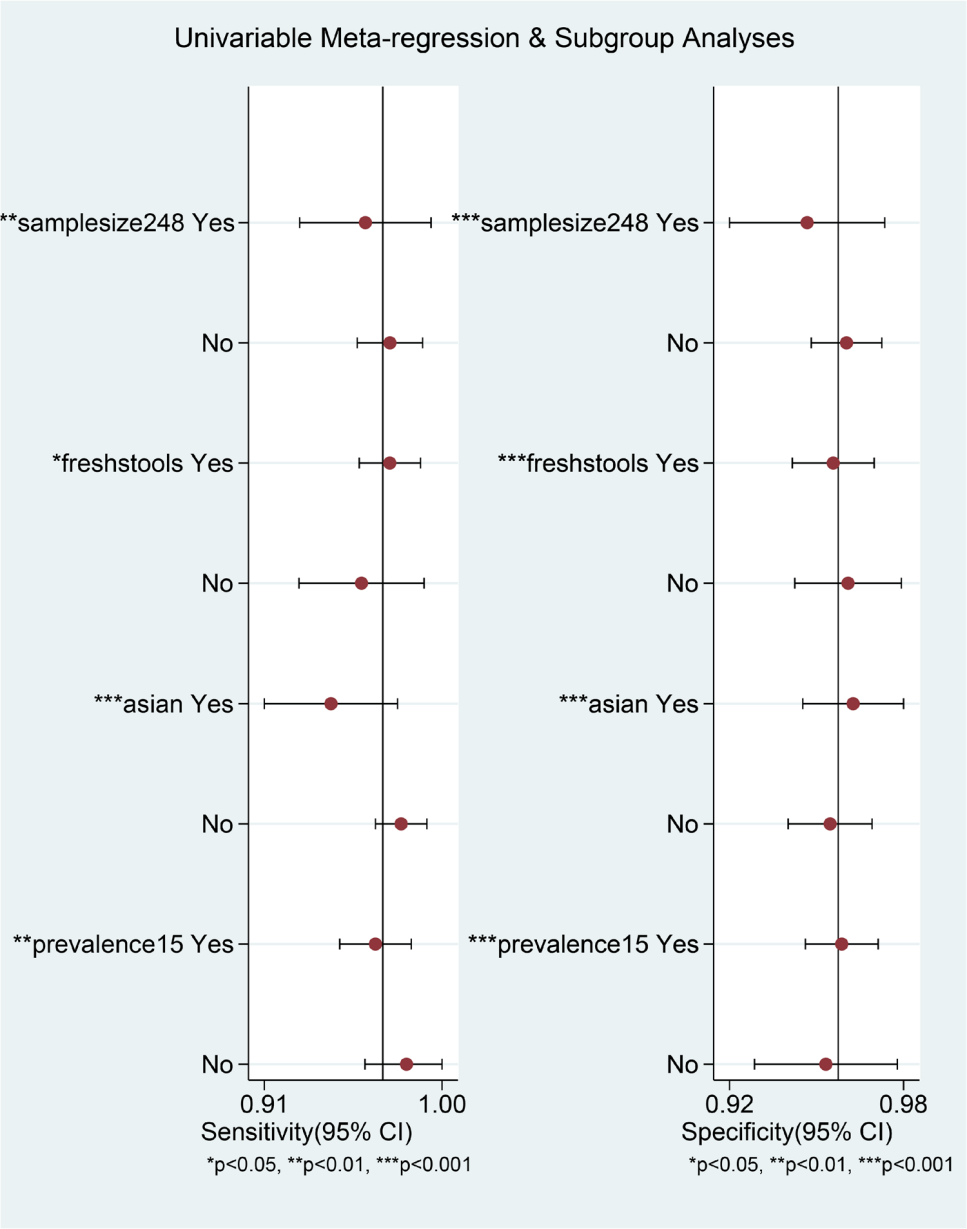
Univariate meta-regression and subgroup analysis for sensitivity and specificity. Factors with asterisk are potential sources of heterogeneity

Meta-regression analyses were performed to further investigate the potential sources of inter-study heterogeneity (Fig. 7). Notably, the results showed that the sample size, sample type, the type of ethnicity, prevalence, and whether blinded all were conspicuous sources of heterogeneity, and most of these variables had a more significant effect on specificity than sensitivity. In addition, subgroup analyses based on these variables were performed; the related parameters of which, including pooled sensitivity, specificity, PLR, NLR, and DOR for each subgroup, are also listed in Table 2.

## Discussion

The incidence and severity of CDI have been increasing significantly worldwide with associated morbidity, mortality, and healthcare costs [41]. A rapid and accurate diagnosis is essential to guide the treatment and to prevent transmission. It has been shown that rapid diagnosis positively impacts on patient’s care by reducing delays in initiation of isolation and treatment for confirmed CDI cases [42]. The Xpert CD assay is now implemented in many countries due to its shorter turnaround time, thus a more effective procedure. The most significant advantage of the Xpert CD assay is its rapidity and simplicity. According to the Society for Healthcare Epidemiology of America and the Infectious Diseases Society of America guidelines, “...PCR testing appears to be rapid, sensitive, and specific and may ultimately address testing concerns. More data on utility are necessary before this methodology can be recommended for routine testing.”

To provide much more evidence-based results for the utility of this assay in routine testing, we performed a meta-analysis to comprehensively evaluate the overall diagnostic accuracy of the Xpert CD assay in detecting CDI compared with reference tests in 2017. Our previous meta-analysis concluded that the Xpert CD assay had good accuracy for detecting CDI (sensitivity 0.97(0.95–0.99); specificity 0.95(0.94–0.96); PLR 21.41(16.66–27.52); NLR 0.03 (0.02–0.05); DOR 762.13(401.82–1445.52); and AUC 0.99(0.97–0.99)). Nonetheless, we found significant heterogeneity for diagnostic parameters among the studies analyzed and the considerable heterogeneity among the results remained unexplained even after subgroup analysis.

Since the publication of our previous report, several newer studies have been published to evaluate the diagnostic accuracy of Xpert CD assay, and four new researches including 1141 cases meet the inclusion criteria for the meta-analysis. We implemented this meta-analysis to offer an up-to-date and comprehensive analysis. A total of 26 independent studies (including 10,493 samples) were finally included in this updated meta-analysis. Moreover, further advantageous features introduced into the analysis methodology included: (1) influence analysis and outlier detection were performed to evaluate the robustness of the tests; (2) meta-regression and subgroup analyses were conducted to evaluate the prospective sources of heterogeneity; and (3) the Deeks’ funnel plot asymmetry test was conducted to assess publication bias.

Since meta-analytic procedures model the summary, estimations are only as trustworthy as the implemented models. Thus, evaluations of model diagnostics (e.g., goodness of fit, influence analysis, outlier detection) are important [43], while there were only two influential outliers through influence analysis and outlier detection. After we excluded the two outliers and then made the same analyses for the leaving studies, we found that the overall results did not change significantly (Table 2), confirming the robustness of our meta-analysis. However, the Deeks’ funnel plot asymmetry test showed that there was statistically significant publication bias (*p* = 0.04).

As with our previous meta-analysis, this up-to-date meta-analysis still showed significant heterogeneity for diagnostic parameters among the studies analyzed. The Spearman correlation coefficient between the logit of sensitivity and logit of 1-specificity was not significant, indicating that the heterogeneity was not caused by threshold/cutoff effect. Since there were variations in sample characteristics and preanalytical and analytical procedures, heterogeneity can be assumed in diagnostic accuracy meta-analyses. However, unlike meta-analyses of treatment studies, there are no generally accepted measures of heterogeneity for diagnostic accuracy in meta-analyses [44]. Meta-regression can be used to identify and screen the main factors of heterogeneity, analyze the sources of heterogeneity, and provide guidance for further data collection in the future. It also provides the basis for future subgroup analysis.

Meta-regression and subgroup analysis in this meta-analysis were conducted to explore potential sources of heterogeneity. The meta-regression results showed that the sample size (> median 248 or < 248), sample type (fresh stool or frozen stool), the type of ethnicity (Asian or Caucasian), and prevalence (> 15% or < 15%) were conspicuous sources of heterogeneity, and we found that most of these variables had a greater influence on specificity than sensitivity in the inter-study heterogeneity (refer Fig. 7). It is important to be aware not only of the sensitivity and specificity of an assay but also of the CDI prevalence in the tested population, as the predictive values and hence the clinical utility of the assays depend on them [45]. Here the demographic characteristics (e.g., race, region) of the subjects in different regions are different, and the research levels in different countries are also inconsistent, so ethnicity (Asian or Caucasian) may be a potential source of heterogeneity. Subgroup analyses based on these variables were performed to test for causes of variations other than threshold effect. There were no significant heterogeneity for PLR (*I*^2^ = 23.39%, *p* = 0.01) and DOR (*I*^2^ = 21.93%, *p* = 0.19) when CDI prevalence of studies greater than 15% were pooled and that for sensitivity (*I*^2^ = 37.05%, *p* = 0.09), NLR (*I*^2^ = 13.8%, *p* = 0.31) and DOR (*I*^2^ = 0.00%, *p* = 0.69) when sample size of studies less than median sample size were pooled. There was moderate heterogeneity for sensitivity (*I*^2^ = 60.16%, *p* = 0.00) and NLR (*I*^2^ = 55.56%, *p* = 0.00) when studies using fresh stools tests were pooled. The results suggested that the CDI prevalence, sample size, and sample type could partly explain the heterogeneity.

The goal of this meta-analysis was to determine whether the Xpert CD assay had good diagnostic accuracy for CDI. This updated analysis showed this assay had “very good” diagnostic accuracy (sensitivity 0.97(0.95–0.98); specificity 0.96(0.95–0.97); PLR 23.81(17.93–31.61); NLR 0.03 (0.02–0.05); DOR 784.85(440.25–1399.16); and AUC 0.99 (0.98–1.00)). The performance of NAATs is commonly assessed using diagnostic accuracy measures for the presence of the *C. difficile* organism (e.g., sensitivity, specificity, PPV, and NPV). However, these measures may not directly link to the clinical definition of CDI or clinical outcomes, and some measures (e.g., PPV and NPV) are dependent on CDI prevalence in the patient population being tested [46]. Obviously, laboratory testing alone without considering the patient’s total clinical features is not appropriate for CDI diagnosis. Therefore, one of the limitations of our study was refined to be based only on the intermediate outcome of diagnostic accuracy for detecting the presence of the toxin genes; due to that, most of the included studies have limited evidence linking laboratory diagnosis with clinical outcomes.

Further, like other NAATs, one of the more important questions concern the clinical utility of Xpert CD assay is that it specifically detects the *tcdB* gene encoding the toxin and not the toxin itself. Therefore, asymptomatic carriers can be misdiagnosed as disease state patients if inappropriate testing is performed. To avoid overdiagnosis and overtreatment of toxigenic CDI by using the Xpert CD assay, it must be strictly limited to diarrheal stool specimen in patients without laxatives. The IDSA guideline states that if patients without laxatives meet the clinical criteria for CDI, and the laboratory does not test formed stools, either a stand-alone NAAT or an algorithm-based approach is acceptable [13]. The ASM guideline also endorses a stand-alone role of NAATs in CDI diagnosis. Indeed, the key to minimizing CDI overdiagnosis is appropriate patient selection, regardless of the diagnostic method used [47]. Preanalytic data are often used by clinicians when deciding if a patient should be tested for the presence of toxigenic *C. difficile*, such as history of antibiotic use or prior hospitalization, more than three times of diarrhea within 24 h, patient age, and residence in long-term care facilities. Consistently, our quality assessment analysis showed that “patient selection” provided the most high-risk bias and high-risk applicability concerns. All the included studies reported that the laboratory tested unformed or liquid stool; however, other details such as history of antibiotic use or prior hospitalization, and other known factors, were not included in the vast majority of diagnostic accuracy comparison studies, which made us fail to analyze the influence of preanalytic indicators on the diagnostic accuracy of Xpert CD assay. Moreover, even though the assay showed “very good” diagnostic accuracy, the absence of preanalytical factors also limits whether this meta-analysis can fully answer the critical question “Does this patient have CDI?” Therefore, it is crucial to note that the preanalytic variables of the clinical presentation should be taken into account with the interpretation of the diagnostic test result in future studies.

In conclusion, NAATs offer the combination of speed, sensitivity, high negative predictive value, and cost-effectiveness when used appropriately [7]. Stand-alone NAAT testing continues to be widely used for CDI. Although the up-to-date meta-analysis showed the Xpert CD assay had good accuracy for detecting CDI, however, it is clear that preanalytical factors are crucial for NAAT specifically. Hence, the diagnosis of CDI must combine patient presentation with diagnostic testing in the future. Low Xpert CD assay cycle threshold could indicate cytotoxicity assay positive patients and those with increased risk of mortality and possibly recurrence [48]. Lower mean quantification cycle values of NAATs could be a predictor of toxin presence in CDI [49]. Therefore, future studies are also needed to focus on the prediction of the disease severity and clinical outcomes. The inclusion of preanalytical parameters and clinical outcomes in study design would provide a more objective evidence base.
